# Directing and Controlling the Business of Nursing

**Published:** 1909-11

**Authors:** Eugene Underhill


### CORRESPONDENCE.

### Directing and Controlling the Business of Nursing.

*Editor Buffalo Medical Journal:*

Sir—For some years it has been apparent to many leading physicians throughout the country, that the medical profession would be obliged to exercise its right and privilege of directing and controlling the business of nursing. This necessity has become still more apparent in recent years by the baneful effects of the so-called "State Registration" movement.

Few physicians can be found who have not had unfortunate experiences with the meddlesome and prescribing nurse. The declaration of many physicians that the state registration movement tends to develop wholesale quackery, and to create a class of insubordinate nurses, with a show of legal authority to apparently justify their claim to equal privilege in directing the affairs of the sick room, is undoubtedly true. The state registration movement has also tended to place the control of nursing in the hands of a few dictorial persons, whose desire seems to be to limit the supply of nurses to hospitals, and to so manipulate and elevate prices, as to prevent the poor and the great middle classes from securing adequate nursing assistance.

The Physicians' National Board of Regents will classify and list all nurses who are willing to pledge themselves to abide by the instructions of the attending physician, and not attempt to play the role of doctor. Four classifications will be made:

1. Commissioned and Official Nurses (Those having completed a two years' course or more in a general hospital or training school.)

2. Approved Nurses. (Those having completed a two years' course in a special hospital.)

3. Attendant Nurses. (Those engaging in nursing, after having had only a theoretical or correspondence course of instruction.)

4. Provisional Nurses. (Those having been engaged in nursing for a year or more, i.e., the so-called practical nurse.)

It is intended to publish and have on file at every County Medical Society and available also to individual physicians, a national calender of nurses, showing classification and credentials. Ample resources have been provided to insure the execution of these plans.

Respectfully,

EUGENE UNDERHILL.

